# Changes in Antioxidant System during Grain Development of Wheat (*Triticum aestivum* L.) and Relationship with Protein Composition under FHB Stress

**DOI:** 10.3390/pathogens9010017

**Published:** 2019-12-23

**Authors:** Valentina Spanic, Marija Viljevac Vuletic, Daniela Horvat, Bojan Sarkanj, Georg Drezner, Zvonimir Zdunic

**Affiliations:** 1 Agricultural Institute Osijek, Osijek, Juzno Predgradje 17, 31000 Osijek, Croatia; marija.viljevac@poljinos.hr (M.V.V.); daniela.horvat@poljinos.hr (D.H.); georg.drezner@poljinos.hr (G.D.); zvonimir.zdunic@poljinos.hr (Z.Z.); 2 Department of Food Technology, University Centre Koprivnica, University North, Trg dr. Žarka Dolinara 1, HR-48000 Koprivnica, Croatia; bsarkanj@unin.hr

**Keywords:** *Fusarium*, gliadins, glutenins, HMW, LMW

## Abstract

Fusarium head blight (FHB) is found in both temperate and semi-tropical regions and causes losses in wheat (*Triticum aestivum* L.) resulting in reduced yield, deteriorated grain quality, and contamination of the grains with mycotoxins, primarily deoxynivalenol (DON). In this study, we focused on the identification of protein components in *Fusarium*-inoculated and non-inoculated wheat samples along with the major antioxidant enzymes that *Fusarium* can encounter during FHB infection process in six winter wheat varieties when FHB symptoms started to occur. Our hypothesis was that wheat antioxidants and H_2_O_2_ may play a role against *Fusarium* infections, consequently preserving protein grain components. Results showed that in more resistant varieties (Olimpija and Vulkan) DON content of inoculated flour was less accumulated and thus the major gluten network was not significantly attacked by *Fusarium* spp. The maximum increase in guaiacol peroxidase (POD) activity in response to FHB treatment was detected in the FHB-resistant varieties Olimpija and Vulkan, while the lowest increase in H_2_O_2_ content was detected in the FHB-susceptible variety Golubica. A particular reduction in the content of both total glutenin and high-molecular-weight glutenin subunits was detected in susceptible wheat varieties after serious artificial infection, along with increased DON accumulation. The defense mechanism in wheat varieties differed, where resistant varieties’ protein composition depended on POD activity as a detoxification agent.

## 1. Introduction

Fusarium head blight (FHB) is one of the most dangerous fungal diseases of wheat. It can cause serious epidemics worldwide. Due to the importance of wheat product consumption, understanding the impact of *Fusarium* infestation on grain characteristics is essential. *Fusarium* infection leads to an incomplete accumulation of grain constituents through mechanical blocking of vascular bundles by mycelium [1] or impaired synthesis of grain components due to the presence of mycotoxins [2]. Although FHB results in quality reductions and yield losses, the major concern for human and animal consumption is the contamination of grains with harmful mycotoxins, including deoxynivalenol (DON), which is the most abundant out of the regulated mycotoxins. The European Union has established maximum levels for DON (Commission Regulation (EC) No. 1881/2006), where unprocessed wheat, cereal flour, bread, and wheat-based foods for infants and young children must not contain more than 1250, 750, 500, and 200 μg kg^−1^ of DON, respectively. DON is known to cause food refusal, vomiting, and depressed immune function resulting in poor weight gain [3]. Furthermore, DON can weaken the immune system of the plants giving the *Fusarium* spp. easier access to nutrients [4]. Usually *Fusarium* symptoms on plants and DON concentrations are very well correlated. The most effective measure to control FHB and reduce mycotoxin contamination is the improvement of resistance to disease and mycotoxin accumulation [5], together with appropriate agronomic practices. Two major types of resistance have been classified for wheat, which includes type I, resistance to initial infection [6], and type II, resistance to spread of symptoms [6]. Further types or components of FHB resistance have been described [7], including resistance to kernel infection, tolerance to yield loss, and resistance to mycotoxin accumulation.

Environmental stress affects plant development and triggers different morphological, physiological, biochemical, and molecular changes in plants. Cells can tolerate a small to moderate amount of oxidative stress by producing non-enzymatic antioxidant molecules and activating enzymes to counteract excess reactive oxygen species (ROS) [8] whose generation is one of the most common plant responses to different stresses [9]. ROS have a dual role as toxic compounds and regulators of antioxidant defense through the ascorbate–glutathione pathway (Halliwell–Asada cycle). Beside the enzymatic part of antioxidative defense, phenolics are considered to be the major contributors to the total antioxidant capacity of cereal grains [10,11] that could play a role in the inhibition of *Fusarium* species and reduction of fungal growth and mycotoxin accumulation.

FHB affects the grain protein content [12] by destroying starch granules, storage proteins, and cell walls and consequently decreases the quality of dough properties. Wheat grain quality is a complex trait determined by the total protein concentration and its composition. In grains, there are two groups of proteins, glutenins (GLU) and gliadins (GLI), which are also major components of gluten. These groups can also be separated based on their molecular weight (ω-, α-, and γ-gliadins; high-molecular-weight (HMW) and low-molecular-weight (LMW) glutenins). These two groups make up 80%–85% of total protein in mature wheat grains. The most important indicators for determining dough elasticity are the HMW glutenin subunits (HMW-GS), which are positively correlated with bread-making quality [13]. Both GLI and GLU can be considerably degraded by *Fusarium* infection [14]. 

Therefore, the aims of this study were (i) to determine enzymatic and non-enzymatic components of antioxidative defense in *Fusarium*-inoculated and non-inoculated ears of six wheat varieties when first FHB symptoms occurred and (ii) to evaluate the potential protective effects of antioxidant defense toward *Fusarium* and consequently DON accumulation and changes in protein components, which can be declared as novel FHB-resistant components [15]. 

## 2. Results and Discussion

In order to extend our knowledge about information on different antioxidant system alterations caused by infection with one of the most important wheat pathogens, we analyzed changes in the activity of antioxidant enzymes and non-enzymatic components involved in defense response when the first symptoms of FHB occurred. Furthermore, we focused specifically on the most toxic and widespread trichothecene DON, which could be the cause of wheat quality reduction by disrupting the normal function of mitochondria and generating ROS [16]. In addition, we tracked the changes in final protein composition in grains responsible for baking quality.

### 2.1. FHB Severity and Incidence

**Figure 1:**
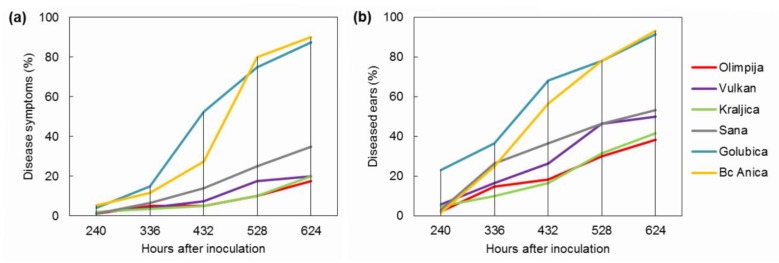
Disease symptoms for (**a**) general resistance and (**b**) diseased ears for type I resistance in inoculated plants for six wheat varieties.

Disease symptoms for the calculation of the area under the disease progress curve (AUDPC) for general resistance started to increase at 10 days after infection (dai), when disease severity for varieties Olimpija, Vulkan, Kraljica, Sana, Golubica, and Bc Anica was 1.25%, 1.75%, 2.0%, 1.25%, 4.25%, and 5.25%, respectively. In the last assessment (624 h after inoculation, hai), the variety Bc Anica had up to 90% *Fusarium* symptoms (Figure 1a). In varieties Golubica and Bc Anica, AUDPC for disease severity was much higher (430.0 and 358.3, respectively), compared with varieties Olimpija and Kraljica, which had less sporulation on the inoculated ears (Table 1). The variety Olimpija had the lowest AUDPC for general resistance (72.5). Olimpija and Kraljica also showed the best resistance against pathogen penetration (AUDPC for type I resistance = 199.9 and 199.1, respectively). First symptoms of the initial infection were visible at 10 dai, when they started to increase up to 38.3% (Olimpija), 49.9% (Vulkan), 41.7% (Kraljica), 53.3% (Sana), 91.7% (Golubica), and 93.3% (Bc Anica) (Figure 1b). These data had already been published for Vulkan, Kraljica, Olimpija, and Golubica in the previous research of Spanic et al. [16], but the present study is used to make additional conclusions. No FHB symptoms were found in the non-inoculated plots. Although Olimpija showed the least FHB symptoms, Vulkan, as the resistant variety, had the lowest amount of DON (Table 1). This could be due to higher resistance to mycotoxin accumulation, a component of FHB resistance [7].

### 2.2. Enzymatic and Non-Enzymatic Components of Antioxidant Defense System

An analysis of variance revealed wide variations for the variety and treatment effects for guaiacol peroxidase (POD), ascorbate peroxidase (APX), catalase (CAT), and polyphenol oxidase (PPO) activity, as well as for hydrogen peroxide (H_2_O_2_), malondialdehyde (MDA), and phenol accumulation, except between varieties for CAT. Interactions were significant for all enzyme activities and non-enzymatic components parameters, except between variety by treatment interaction for phenols (Table 2).

**Figure 2:**
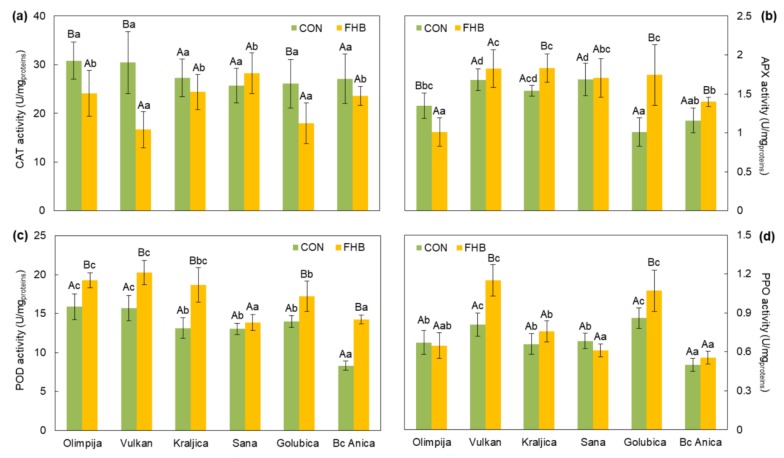
CAT, APX, POD, and PPO activity in non-inoculated (CON) and inoculated (FHB) plants of six wheat varieties. Values are means of five replications ± standard error (SE). Capital letters indicate significantly different values (according to Fisher’s LSD test (*p* ≤ 0.05) in different treatments (non-inoculated and inoculated treatments). Lower case letters indicate significantly different values (according to Fisher’s LSD test (*p* ≤ 0.05) among different varieties under the same treatment.

In the present study, the varieties Olimpija, Vulkan, and Golubica had significantly lower CAT values in the inoculated plants, compared to non-inoculated plants (Figure 2).

The activity of APX increased with the appearance of symptoms in Kraljica, Golubica, and Bc Anica in inoculated plants, compared to non-inoculated plants, while APX activity declined in Olimpija in inoculated FHB plants, compared to non-inoculated plants (Figure 2). 

An increase in POD activity, in response to *Fusarium* inoculation, compared to non-inoculated plants, was detected in all varieties, except Sana (Figure 2). During the stage of post-infection, inoculated ear tissues of the resistant variety Vulkan and the susceptible variety Golubica showed significantly higher PPO activity, compared to non-inoculated ears (Figure 2). 

**Figure 3:**
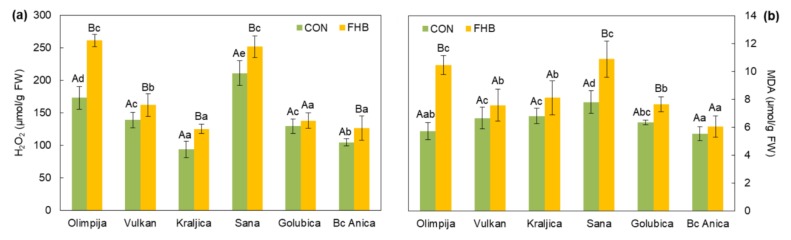
H_2_O_2_ and MDA content in non-inoculated (CON) and inoculated (FHB) plants of six wheat varieties. Values are means of five replications ± standard error (SE). Capital letters indicate significantly different values according to Fisher’s LSD test (*p* ≤ 0.05) in different treatments (non-inoculated and inoculated treatments). Lower case letters indicate significantly different values according to Fisher’s LSD test (*p* ≤ 0.05) among different varieties under the same treatment.

An increase in H_2_O_2_ was observed in both resistant and susceptible varieties, except Golubica, after *Fusarium* inoculation (Figure 3). In a study by Audenaert et al. [4], high DON concentrations were shown to trigger H_2_O_2_ evolution. DON was shown to be a virulence factor for *Fusarium graminearum* infecting wheat by promoting the spreading of the pathogen [17]. The highest H_2_O_2_ content among infected plants was found in the ears of the resistant variety Olimpija and the susceptible variety Sana. Peroxide accumulation in resistant Olimpija possibly occurred as an effect of the signaling role of H_2_O_2_, whereas in Sana H_2_O_2_ overload could be a consequence of deficient functioning of the antioxidant system. The activation of H_2_O_2_-mediated defense responses comprising phenols and peroxidases might come too late for susceptible varieties to protect itself against *Fusarium* in our research.

The susceptible varieties Sana and Golubica and resistant Olimpija showed significantly higher MDA content in inoculated ear tissues, compared to non-inoculated ears (Figure 3). Furthermore, we assumed that DON exposure increased peroxidation and decreased antioxidant activity in susceptible varieties, but in the resistant variety there was probably another mechanism which increased MDA. Yang et al. [18] had already reported about oxidative stress and its important role in the toxicity of trichothecenes. In Olimpija, smaller amount of DON could generate H_2_O_2_ that induced lipid peroxidation, which in turn leads to changes in membrane integrity, cellular redox signaling, and antioxidant status. 

The decline in CAT activity was correlated with the increase of H_2_O_2_ in FHB-resistant varieties (Olimpija and Vulkan) after inoculation with *Fusarium*. This could be due to a connection with DON resistance, as Olimpija and Vulkan had the lowest amount of DON among other varieties. Mycotoxin contamination is also related to the capacity of plant tissues in reducing mycotoxin accumulation, where host metabolites are able to interfere with mycotoxin biosynthesis [19]. Previously, it was concluded that free phenolic acids are evidently involved in the DON resistance mechanism in maize, whereas their role toward fumonisin contamination was not elucidated under field conditions, implying that components other than phenolic acids may be responsible for this latter type of resistance [20]. In the present investigation, the AUDPC for general resistance showed significant and positive correlation with DON (Spearman’s ρ: 0.83, *p* < 0.05), where a strong positive correlation was observed with AUDPC for type I resistance (Spearman’s ρ: 0.94, *p* < 0.01) (Table 3). The selection for reduced FHB symptoms should lead to a correlated selection response in low DON content in grains [21].

On the other hand, Olimpija and Vulkan exhibited high POD activity indicating that POD in those genotypes had a major role in ROS detoxification providing FHB resistance. Similarly, an increase in POD activity was observed in the research of Lanubile et al. [22] in the resistant maize genotype, both for control and inoculated kernels which were inoculated with *Fusarium proliferatum*, *Fusarium subglutinans*, and *Aspergillus flavus.* Contrary to that research, a decrease in CAT was observed in the resistant varieties Olimpija and Vulkan. In a study by Hassanein et al. [23], it was concluded that the host cell under pathogenesis might accelerate the terminal respiratory pathway, which may lead to an increase in CAT activity. 

**Figure 4:**
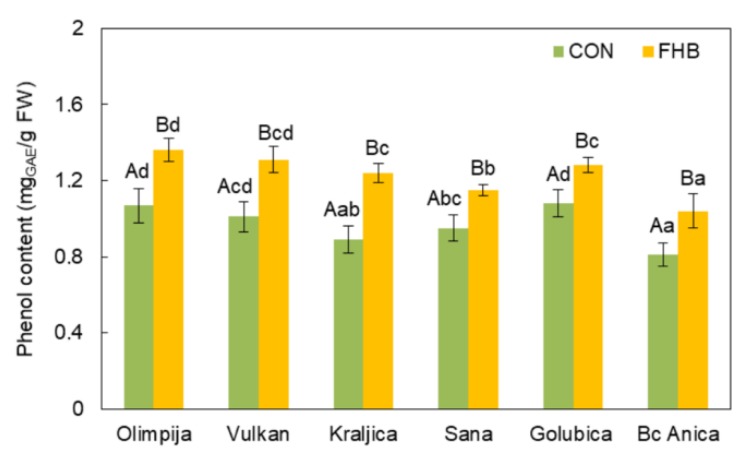
Phenol content in non-inoculated (CON) and inoculated (FHB) plants of six wheat varieties. Values are means of five replications ± standard error (SE). Capital letters indicate significantly different values according to Fisher’s LSD test (*p* ≤ 0.05) in different treatments (non-inoculated and inoculated treatments). Lower case letters indicate significantly different values according to Fisher’s LSD test (*p* ≤ 0.05) among different varieties under the same treatment.

**Figure 5:**
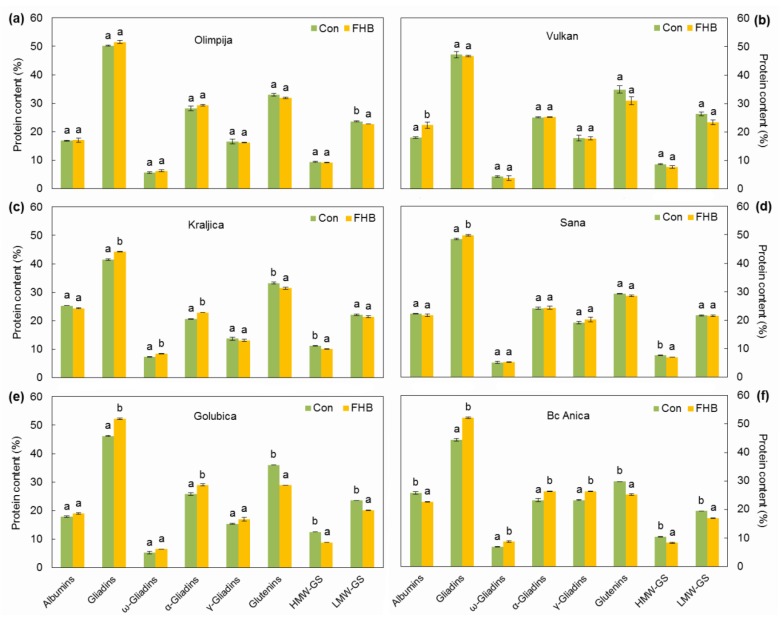
Percentage of albumins and globulins; gliadins; ω-, α-, and γ-gliadins; glutenins; high molecular weight (HMW)-glutenin subunits (GS), and low molecular weight (LMW)-GS in non-inoculated (CON) and inoculated (FHB) samples. Letters indicate significantly different values according to Fisher’s LSD test (*p* ≤ 0.05) in different treatments (non-inoculated and inoculated treatments) for each variety and each protein composition.

Phenolics are considered to be the major contributors to the total antioxidant capacity of cereal grains. Moreover, it was concluded that they are the most important components in winter wheat resistance to fungal diseases [24]. In the current research, the varieties Golubica and Vulkan exhibited higher PPO activity in *Fusarium*-inoculated plants compared to non-inoculated plants, despite the presence of increased phenol content in the ears of inoculated plants in all varieties (Figure 4). The accumulation of phenols in the infected tissues might come from the surrounding healthy leaves in order to resist the advancement of the pathogen toward the healthy cells [25]. In addition, total phenols significantly increased in *Fusarium*-inoculated plants compared to non-inoculated controls, which is in accordance with the research of Hassanein et al. [23] where total phenols increased throughout the experimental period. In a previous research by Spanic et al. [26], it was concluded that FHB-resistant wheat varieties showed rapid induction of ascorbate peroxidase (APX) and polyphenol oxidase (PPO) activity in the early stages after infection (3 hai). This is in accordance with the research of Mohammadi and Kazemi [27] where a 3-fold increase in polyphenol oxidase activity was detected in the resistant wheat varieties after inoculation with *F. graminearum* compared with the non-inoculated controls. The most susceptible variety (Bc Anica) had the lowest amount of phenol content and PPO activity among other varieties, which indicated the poor effectiveness of non-enzymatic antioxidative components against pathogen attack. Due to the role of polyphenols as secondary plant metabolites that play a role in the protection of plants against pathogens, they were higher in varieties with better preserved protein quality (Olimpija, Vulkan, and Kraljica) (Figure 5). In a previous research by Boyacioǧlu and Hettiarachch [28], infection with *F. graminearum* decreased the proportion of water-extractable protein (albumin) and storage protein (glutenin) by 33% and 80%, respectively, in infected wheat. 

In the current research, there was a significant increase in phenols, POD, and H_2_O_2_ for all wheat varieties in the *Fusarium*-inoculated group compared to non-inoculated plants, except for POD in Sana and H_2_O_2_ in Golubica. The lack of significant accumulation of H_2_O_2_ in inoculated Golubica plants, compared to non-inoculated plants, could be the reason for the strong decrease in protein quality and high FHB severity. In the research of Spanic et al. [26], it was concluded that in resistant wheat varieties, H_2_O_2_ level was higher in the inoculated ears compared to non-inoculated plants in the early stages of disease (24 hai). A similar result was previously reported by Sorahinobar et al. [29]. An increased amount of H_2_O_2_ could be toxic to pathogens and can lead to a hypersensitive response of plants resulting in cell death, which will prevent the further spread of pathogens. An increase in APX activity in *Fusarium*-inoculated Kraljica, Golubica, and Bc Anica plant varieties could not detoxify H_2_O_2_, which led to decreased glutenins and HMW subunits. In resistant Olimpija, APX activity decreased in inoculated plants compared to non-inoculated controls, which led us to the conclusion that overall H_2_O_2_ was removed by POD rather than APX.

### 2.3. Protein Composition

*Fusarium* inoculations did not significantly change the proportion of albumins and globulins in the grain samples of four varieties (Olimpija, Kraljica, Sana, and Golubica), while the proportion was significantly increased up to 24.4% in Vulkan infected with *Fusarium* compared to non-inoculated plants, in contrast to Bc Anica which decreased the proportion of albumins and globulins up to 12.1% (Figure 5). Albumins and globulins varied from 16.6%–25.8% of total flour protein in both inoculated and non-inoculated plants. The data for protein components in the grain samples had already been published for Vulkan, Kraljica, Olimpija, and Golubica in a previous research [16], but the present study makes additional conclusions. 

Gliadin level significantly increased in infected plants compared to non-inoculated plants in four varieties (Kraljica, Sana, Golubica, and Bc Anica). The total gliadin content ranged between 41.3% and 52.5% in both inoculated and non-inoculated plants. In infected Bc Anica plants, ω-, α-, and γ-gliadins were significantly increased compared to non-inoculated plants. A similar increase was observed in infected plants for ω- and α-gliadins in Kraljica and α-gliadins in Golubica (Figure 5). Within the gliadins, the most affected subfractions were ω-gliadins, which were increased up to 26.9% compared to non-inoculated plants. At the same time, there was one variety (Vulkan), in which a decrease of 12% in ω-gliadins was observed. α-Gliadins were increased in all varieties in infected plants compared to non-inoculated controls (up to 13.3%, Bc Anica). A non-significant increase in γ-gliadins was observed in Bc Anica (19.7%), Golubica (10.9%), and Sana (5.7%). 

*Fusarium* infection had a significant effect on the percentage of glutenins, which showed a significant reduction of 5.4%, 15.6%, and 19.9% in Kraljica, Bc Anica, and Golubica, respectively, compared to non-inoculated controls, accompanied by a decrease in HMW subunits (9.9%, 20.3%, and 29.8%, respectively), including Sana (8.8%). These varieties had higher DON accumulation (Table 1). It was previously concluded that quality reduction was a result of contamination by trichothecene mycotoxins produced by *Fusarium* species [30]. LMW-GS subunits were not as largely affected as HMW-GS, but Olimpija, Golubica, and Bc Anica showed a significant decrease (4.1%, 14.6%, and 12.8%, respectively) (Figure 5).

Out of albumins and globulins, α-amylase/trypsin inhibitors, serpins, and purothionins may serve as nutrient reserves for embryo development or inhibitors of insects and pathogens prior to germination [31]. Golubica had the highest decrease in HMW-GS in the inoculated group, but at the same time, it had increased albumins and globulins post-infection. HMW-GS was also reduced in Bc Anica and Kraljica in the inoculated group. Due to this observation, we can hypothesize that albumins and globulins might play a defense role in FHB pathogen attack in the latter stages of plant maturity, which still needs to be investigated in the future. In wheat maturity, the rapid loss of water content may be due to a blockage of water in flow by lipid deposition in the chalaza. According to the increased MDA content in Sana, Golubica, and Olimpija, this blockage did not occur properly and APX activity was elevated in Golubica in the inoculated group compared to non-inoculated plants. In contrast, APX increased due to pathogen attack in Olimpija. Sana also had reduced HMW-GS due to unchanged POD activity and higher MDA content in *Fusarium*-infected ears compared to non-inoculated ears. Generally, the highest reduction after *Fusarium* inoculation among protein components was noticed in HMW-GS in Golubica (29.8%) and Bc Anica (20.3%) compared to non-inoculated controls, which is in accordance with the research of Eggert et al. [32], where gluten digestion by *F. graminearum* proteases showed a preference for glutenins compared to gliadins, whereas the HMW-GS subfraction was the most affected.

In contrast to gliadins which were increased in infected plants, the total glutenin content (particularly HMW-GS) was reduced in *Fusarium*-infected plants compared to non-inoculated controls in this investigation, which is in accordance with previous research [14]. Among protein types, in the current research, ω-gliadins were highly affected, which is slightly different from the results of research reported by Horvat et al. [14], where α-GLI was the highest affected protein type. Within the gliadins, the most affected subfractions were ω1.2-gliadins which were reduced by 45% and γ-gliadins which were reduced by 26%, compared to non-inoculated controls [32]. An influence on protein fractions, such as an increase in gliadin content and a reduction in glutenin content, has also been recorded [33]. Baking parameters stayed nearly unchanged, although *Fusarium* infection levels and DON contents were high [34]. The significant effect of disease on HMW-GS might be due to the poor or intermediate richness of these two components with sulfur-containing amino acids and abundance of glutamine and proline [31], which gives them an advantage in nitrogen storage, which could be a nutrient supply for the pathogen. Therefore, more HMW-GS could attract more pathogen biomass, resulting in a higher susceptibility of plants. 

The list of resistance components must be completed with aspects of resilience of rheological properties, including protein composition as a baking-quality determinant [15], which was determined in the current research. In this study, some wheat varieties had good grain resistance to FHB, which may be due to the different antioxidant activities. FHB does not affect the grain biosynthesis processes, but impacts the transport of assimilates caused by changes in grain composition claiming that the resilience of rheological properties following FHB infection pressure is an additional component of grain resistance to the disease [15]. The breakdown of HMW-GS may explain the observed decrease in dough quality and baking performance after *Fusarium* infection of wheat [32]. The reduced glutenin content may be due to the degradation activity of *Fusarium* proteases [35]. 

## 3. Materials and Methods 

### 3.1. Inoculum Production

The inoculum consisted of two different *Fusarium* species (1:1). *Fusarium culmorum* strain (IFA 104), DON chemotype and highly aggressive, was obtained from the Institute of Biotechnology in Plant Production, IFA-Tulln, Austria. *F. graminearum* was isolated from wheat kernels in a field in eastern Croatia. To produce macroconidia of *F. culmorum*, a mixture of wheat and oat grains (3:1 by volume) was used. The inoculum of *F. graminearum* was prepared by using the “bubble breeding” method with a liquid mung bean medium. Final concentration of conidial suspensions of both the strains was set to 1 × 10^5^ mL^–1^. Concentrated spore suspensions were diluted in 100 L of water before inoculation. For inoculation, 100 mL suspension per square meter was used.

### 3.2. Field Trials

**Figure 6:**
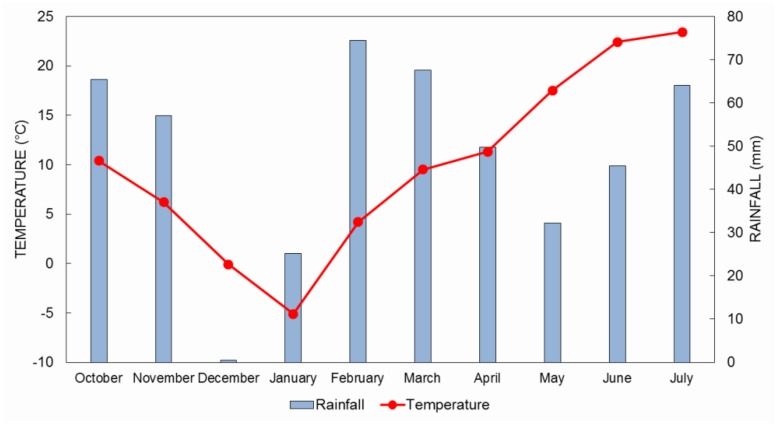
Rainfall and average temperatures during the vegetation season 2016/2017 at Osijek.

The field trial was set up at the Agricultural Institute Osijek (45°32′N, 18°44′E) where the soil type is eutric cambisol. The average annual precipitation during the vegetation period in 2016/17 was 482 mm and the average annual temperature was 10 °C (Figure 6). The experimental plot area was 7.56 m^2^, where one treatment (non-inoculated and artificially inoculated) was replicated. Six winter wheat varieties (Table 4) were artificially inoculated during the flowering stage using a tractor back-sprayer in the late afternoon and repeated two days later (12 and 15 May 2017). To maintain moisture at ear level, water was sprayed a few times during the day with the tractor back-sprayer. For assessing disease severity, we calculated two parameters (general resistance, GR and type I resistance, T1). Disease assessment began with the appearance of the first symptoms 10 days after inoculation, followed by four consecutive scores at intervals of four days which were used to calculate the area under the disease progress curve (AUDPC). The percentage of bleached spikelets (disease intensity) per plot was estimated according to a linear scale (0%–100%). FHB intensity per plot was taken as a measure for general resistance. Disease incidence (percentage of diseased ears per plot) was used as a measure for type I resistance.

### 3.3. Enzyme Activity

For enzyme extraction, five replicates were taken from the non-inoculated and inoculated plants at the time when first symptoms of FHB were visible (25 May 2017), 10 days after inoculation. Each sample consisted of five ears. Ear tissue was ground into a fine powder with liquid nitrogen in the presence of polyvinylpyrrolidone (PVP) using a pestle and mortar. About 0.2 g of ear powder was extracted with 1 mL of 50 mM potassium phosphate buffer (pH 7.0) with 5 mM ascorbic acid and 0.1 mM EDTA. After centrifugation for 15 min at 14,000× *g* and 4 °C, re-extraction with 1 mL of the same buffer was performed and the joint supernatant of crude protein extract was taken for enzyme assays. The protein concentration was determined according to Bradford [36] using bovine serum albumin as a standard. All spectrophotometric analyses were performed on the spectrophotometer Specord 200 (Analytic Jenna), refrigerated centrifuge Universal 320R (Hettich), and thermomixer 5436 (Eppendorf).

#### Measurements of Enzyme Activities

Guaiacol peroxidase (POD; EC 1.11.1.7) activity was determined according to the method described by Siegel and Galston [37]. The reaction mixture consisted of 5 mM guaiacol and 5 mM hydrogen peroxide in 0.2 M phosphate buffer (pH 5.8). The enzymatic reaction was started by the addition of 25 µL of crude protein extract in 975 µL of reaction mixture. Guaiacol peroxidase activity was determined as an increase in absorbance at 470 nm over 2 min and expressed as U/mg_proteins_, where Unit present 1 µM guaiacol oxidized per minute. Ascorbate peroxidase (APX; EC 1.11.1.11) activity was determined according to Nakano and Asada [38]. The reaction mixture consisted of 955 µL 50 mM potassium phosphate buffer (pH 7.0) with 0.1 mM EDTA, 10 µL 25 mM ascorbic acid and 25 µL of crude protein extract. The enzymatic reaction was initialized by adding 10 µL of 12 mM H_2_O_2_ in reaction mixture and decrease in the absorbance at 290 nm was monitored over 2 min. Ascorbate peroxidase activity was expressed as U/mg_protein_, where U (unit) represents 1 µM of oxidized ascorbate per minute. Catalase (CAT, EC 1.11.1.6) activity was measured by the method of Aebi [39] using a 950 µL reaction mixture consisting of 50 mM potassium phosphate buffer and 5 mM H_2_O_2_. The decrease in absorbance at 240 nm was monitored over 1 min after the initiation of reaction by 50 µL of crude protein extract. Catalase activity was expressed as unit (µM of H_2_O_2_ decomposed per minute) per mg protein (U/mg_protein_). Polyphenol oxidase (PPO; EC 1.14.18.1) activity was determined as the rate of oxidation of pyrogallol to *o*-quinones at 40 °C [40]. The increase in absorbance was recorded at 430 nm during 2 min after the initiation of enzymatic reaction by the addition of 15 µL of crude protein extract to 2.2 mL of reaction mixture consisting of 2 mL of 100 mM potassium phosphate buffer (pH 7.0) and 0.2 mL of 100 mM pyrogallol. Polyphenol oxidase activity was expressed as U/mg_protein_, where U (unit) represents 1 µM of pyrogallol oxidized per minute.

### 3.4. Determination of MDA Content and H_2_O_2_ Concentration

The determination of malondialdehyde (MDA) and H_2_O_2_ content was performed using the same homogenized tissues as for enzyme activity. The extraction with 2 mL of 0.1% trichloroacetic acid (TCA) was performed from 400 mg of powdered ear tissue. After 10 min, the homogenate was centrifuged (15 min, 12,000× *g*, 4 °C) and the supernatant was used for MDA and H_2_O_2_ content measurement. MDA, as the decomposition product of polyunsaturated fatty acids of biomembranes, was determined by the thiobarbituric acid (TBA) reaction [41]. The reaction mixture consisted of 0.5 mL supernatant and 1 mL of 0.5% TBA in 20% TCA and it was heated at 95 °C for 30 min and then quickly cooled in an ice bath. After centrifugation (15 min, 14,000× *g*, 4 °C), the absorbance of the supernatant was recorded against blank (0.5% TBA in 20% TCA) at 532 nm. Correction at 600 nm for non-specific turbidity was subtracted. MDA content was expressed as µmol per gram of fresh weight (µmol/g) using an extinction coefficient of 155 mM^−1^ cm^−1^. H_2_O_2_ concentration was quantified according to Velikova et al. [42]. The reaction mixture consisted of 0.5 mL of supernatant, 0.5 mL of 10 mM potassium phosphate buffer (pH 7.0), and 1 mL of 1 M potassium iodide (KI). After incubation for 20 min at room temperature in darkness, the absorbance of the reaction mixture was read at 390 nm. H_2_O_2_ content was determined using H_2_O_2_ as a standard and expressed as µmol per gram of fresh weight (µmol/g).

### 3.5. Determination of Phenol Content

The extraction of total phenol content was performed on 0.25 g of ear tissue powdered by liquid nitrogen in a mortar by addition of 2.5 mL of 96% ethanol. After extraction for 30 min in an ultrasound bath at 80 °C, the homogenate was centrifuged (15 min, 12,000× *g*, room temperature) and the supernatant was collected and used for total phenol determination by the modified Folin–Ciocalteu method [43]. In brief, 20 µL of supernatant was mixed with 1.58 mL of dH_2_O and 0.1 mL of Folin–Ciocalteu reagent (1:1; *v*/*v* diluted with water). After 5 min, 0.3 mL of sodium carbonate solution (20%; *w*/*v* diluted with water) was added. The homogenized reaction mixture was placed for 30 min in a dark place at room temperature, after which absorbance reading at 765 nm was taken in a spectrophotometer (Specord 200, Analytic Jenna). The content of total phenol (PHE) was expressed as mg of gallic acid equivalents (GAE) per gram based on a gallic acid calibration curve.

### 3.6. Protein Extraction and Characterization

The extraction of wheat proteins was done according to the stepwise quantitative procedure of Wieser et al. [44]. Whole meal flour (100 mg) was extracted stepwise with 0.4 M NaCl (albumins and globulins, AG), 50% 1-PrOH (GLI), and 50% 1-PrOH + 2 M urea + 0.05 M of Tris-HCl (pH = 7.5) + 1% DTT (GLU). Protein separation was carried out using the Perkin Elmer LC 200 chromatograph controlled by the TotalChrom software (Perkin Elmer Instruments, Waltham, USA) on a Discovery BIO Wide Pore C18 column (300 Å pore size, 5 μm particle size, 4.6 × 150 mm i.d.) (Sigma-Aldrich Chemie GmbH, Taufkirchen, Germany). The mobile phase consisted of millipore water with 1% trifluoroacetic acid (*v*/*v*) (A) and acetonitrile with 1% trifluoroacetic acid (*v*/*v*) (B). The column temperature was 50 °C and injection volume was 20 µL. The gradient elution profile was as follows: from 24%–54% B in 30 min, isocratic at 90% B for 5 min, returning to the initial conditions in 5 min, and column equilibration in 5 min. The flow rate was controlled at 1.0 mL/min. The peaks were detected at 210 nm with a photodiode array detector. The peak areas under AG, GLI, and GLU chromatograms were summed and used as a direct measure of total content of extractable wheat proteins and consequently, the proportions (%) of protein fractions and single protein types were calculated [44].

### 3.7. Deoxinivalenol (DON) Analyses

The determination of DON with the LC-MS/MS method in wheat grains in *Fusarium*-inoculated and non-inoculated samples was performed according to the method used by Spanic et al. [45,46]. 10 g of wheat grain was ground by IKA M20 (IKA, Staufen, Germany). DON was extracted with 40 mL of acetonitrile:water (84:16, *v*/*v*). The mixture was stirred for 3 min at high speed in a Waring LB10S blender (Waring, East Windsor, NJ, USA). The extract was filtered through Whatman No. 1 filter paper followed by a glass microfiber filter (934-AH). Following filtration, an solid-phase extraction (SPE) column purification was performed by the addition of 15 mL of extract to the SPE column (MultiSep 226 AflaZon+ Multifunctional Columns, RomerLabs, Tulln, Austria). After purification on SPE columns, an aliquot of 6 mL of clean-up solution was dried under a gentle stream of high purity nitrogen (5.0) (Messer, Osijek, Croatia) at 50 °C. The residue was re-dissolved in 400 µL of a mobile phase, transferred into vials, and 20 µL was injected into the LC-MS/MS system. A Perkin Elmer Series 2000 binary pump with auto sampler was combined with API 2000 triple quadrupole MS (SCIEX) for analysis. The Ascentis Express C-18 column was used for mycotoxin separation (150 × 2.1 mm; with 2.7 µm particle size). The column was heated to 45 °C, eluent A was 10 mM formic acid, and eluent B was 10 mM of formic acid in methanol (pH of both the eluents was set to 3.8 with ammonium hydroxide). The gradient started with 80% of eluent A, that was decreased to 50% within 10 min, followed by 20% in the next 5 min, and then to 0% until 16 min; 100% of eluent B was held for 10 min, followed by equilibration to starting conditions for 4 min. The flow rate was set to 200 µL min^−1^. The MS/MS analysis was performed by using an electrospray ionization (ESI) source in both (positive and negative) modes, in two separate runs. The ion spray voltage was set to −4500 V in the negative mode and 5500 V in the positive mode. Nitrogen was used as an ion source and a collision gas. Results were analyzed with the Analyst software version 1.4.2.

### 3.8. Statistical Analysis

Statistical analysis was done using analysis of variance (ANOVA) followed by Fisher’s LSD test (α = 0.05) and Spearman correlation coefficient by Statistica version 12.0 (Statsoft Inc., Tulsa, USA). The reported data for antioxidant and protein parameters represent the mean ± standard error (SE).

## 4. Conclusions

DON was able to induce plant H_2_O_2_ production in both FHB-resistant and FHB-susceptible wheat varieties, which induced increased lipid peroxidation in susceptible wheat varieties. A reduction in CAT activity during *Fusarium* inoculation in resistant varieties was observed in this study, accompanied by an increase in POD activity. The protein components responsible for wheat quality of Vulkan and Olimpija were unchanged in inoculated samples, which could be due to high POD activity through rapid removal of H_2_O_2_ and PPO activity which could participate in the detoxification of phenolic acids induced by the pathogen attack. Our results showed that, under higher pressure of FHB infestation, the content of glutenins decreased in more FHB-susceptible wheat varieties inoculated with *Fusarium* than in non-inoculated plants. FHB caused a reduction in glutenin and HMW glutenin subunits, which are indicators of bread-making quality, as a result of contamination by DON.

## Figures and Tables

**Table 1:** Area under the disease progress curve (AUDPC) for general resistance and type I resistance (initial infection) to Fusarium head blight (FHB) and deoxynivalenol (DON) accumulation for six wheat varieties.

Variety	AUDPC for General Resistance	AUDPC for Type I Resistance	Resistance/Susceptibility	DON µg kg^−1^
Kraljica	75.5	199.1	R	1424
Olimpija	72.5	199.9	R	588
Vulkan	90.8	280.8	R	755
Sana	145.3	333.3	MS	2205
Golubica	430.0	647.5	S	3308
Bc Anica	358.3	471.6	S	2299

R, resistant; MS, moderately susceptible; S, susceptible; DON, deoxynivalenol.

**Table 2:** Analysis of variance for different enzymatic and non-enzymatic antioxidative components.

	Df	MS							
Source of Variation		POD	APX	CAT	PPO	MDA	H_2_O_2_	Proteins	Phenols
Variety (V)	5	64.60 ***	0.6070 ***	43.45 ns	0.33974 ***	14.066 ***	27,360 ***	9.591 ***	0.11683 ***
Treatment (T)	1	229.67 ***	0.4950 ***	437.51 ***	0.15504 ***	58.885 ***	18,891 ***	13.039 ***	1.04280 ***
V*T	5	8.57 ***	0.3092 ***	76.18 **	0.05877 ***	6.549 ***	1950 ***	4.850 ***	0.00831 ns
Error	48	1.83	0.0415	18.33	0.00801	0.673	195	0.774	0.00435

***,**,* = significant at *p* < 0.001, 0.01, and 0.05, respectively; ns = not significant (*p* > 0.05). POD, guaiacol peroxidase; APX, ascorbate peroxidase; CAT, catalase; PPO, polyphenol oxidase; MDA, malondialdehyde; H_2_O_2_, hydrogen peroxide; Df, degrees of freedom; MS, mean sum of squares.

**Table 3:** Correlation analysis between AUDPC for general resistance, AUDPC for type I resistance, and DON accumulation in the grains of six wheat varieties.

	AUDPC for GR	AUDPC for T1	DON Accumulation	
AUDPC for GR	1			
AUDPC for T1	0.94 **	1		
DON Accumulation	0.83 *	0.77	1	

**, Significant at 0.01; *, significant at 0.05; GR, general resistance; T1, initial (type I) resistance.

**Table 4:** Origin and year of release of six investigated winter wheat varieties.

Varieties	Origin	Year of Release
Kraljica	HR, AIO	2010
Olimpija	HR, AIO	2009
Vulkan	HR, AIO	2009
Sana	HR, BC	1983
Bc Anica	HR, BC	2010
Golubica	HR, AIO	1997

HR, Croatia; AIO, Agricultural Institute Osijek; Bc, BC Institute.
